# Nontraditional Risk Markers for Incident Coronary Artery Calcium Among Persons ≥65 Years of Age

**DOI:** 10.1016/j.jacadv.2023.100755

**Published:** 2023-12-19

**Authors:** Alexander C. Razavi, Omar Dzaye, Erin D. Michos, Matthew J. Budoff, Norrina B. Allen, Joao A.C. Lima, Joseph F. Polak, Wendy S. Post, Khurram Nasir, Roger S. Blumenthal, Laurence S. Sperling, Michael J. Blaha, Seamus P. Whelton

**Affiliations:** a Center for Heart Disease Prevention, Emory University School of Medicine, Atlanta, Georgia, USA; b Ciccarone Center for the Prevention of Cardiovascular Disease, Johns Hopkins University School of Medicine, Baltimore, Maryland, USA; c Division of Cardiology, Department of Medicine, Johns Hopkins University School of Medicine, Baltimore, Maryland, USA; d Lundquist Institute, Harbor-UCLA Medical Center, Torrance, California, USA; e Department of Preventive Medicine, Northwestern University Feinberg School of Medicine, Chicago, Illinois, USA; f Department of Radiology, Tufts Medical Center, Boston, Massachusetts, USA; g Division of Cardiovascular Prevention and Wellness, Houston Methodist DeBakey Heart & Vascular Center, Houston, Texas, USA

**Keywords:** age, aged, atherosclerosis, coronary artery calcium, risk factors

## Abstract

**Background:**

The initiation of coronary artery calcium (CAC) is an important physiologic milestone associated with increased cardiovascular disease risk. However, traditional risk factors (RF) do not perform well for predicting incident CAC among the 54 million older U.S. adults.

**Objectives:**

The authors sought to assess the association between nontraditional cardiovascular disease RF and incident CAC in older persons.

**Methods:**

There were 815 MESA (Multi-Ethnic Study of Atherosclerosis) participants ≥65 years of age who had CAC = 0 at Visit 1 and a follow-up CAC scan. Multivariable adjusted Cox hazards ratios (aHR) and C-statistics were calculated to examine the association of nontraditional RF with incident CAC.

**Results:**

The mean age was 70.2 years and 67% were women. The median follow-up time to repeat CAC scan was 3.6 years (IQR: 2.6-9.2 years) and 45% of participants developed incident CAC. Albuminuria (aHR: 1.50, 95% CI: 1.07-2.09), carotid plaque (aHR: 1.32, 95% CI: 1.04-1.66), and thoracic aortic calcification (TAC) (aHR: 1.38, 95% CI: 1.10-1.75) were significantly associated with incident CAC, while higher levels of nontraditional RF including apolipoprotein-B, lipoprotein(a), high-sensitivity troponin T, and N-terminal pro-brain natriuretic peptide were not. When added to demographics, albuminuria, carotid plaque, and TAC provided a greater C-statistic improvement (+0.047, *P* = 0.004) vs all traditional RF combined (+0.033, *P* = 0.05).

**Conclusions:**

Among nontraditional RF and measures of subclinical atherosclerosis, only albuminuria, carotid plaque, and TAC were significantly associated with incident CAC in persons ≥65 years of age. Identification of albuminuria or extracoronary atherosclerosis may help guide the timing of repeat CAC scoring in older persons with baseline CAC = 0.

The average life expectancy in the United States is 79 years of age^1^ and the number of individuals aged ≥65 years is projected to double to more than 80 million by 2040.^2^ There is a very high burden of traditional risk factors among older persons, two-thirds of whom have hypertension and/or dyslipidemia.^3^ In addition, older persons have the highest absolute rate of cardiovascular disease (CVD). However, the relative strength of association between traditional risk factors and CVD events is weaker among older persons,^4^ which is due in part to the increased prevalence of traditional risk factors, survival bias, and increase in competing risk from non-CVD causes.^5^

Coronary artery calcium (CAC), measured via noncontrast computed tomography, directly quantifies an individual’s calcified subclinical coronary atherosclerotic burden and is strongly predictive of CVD risk.^6^ The initiation of CAC represents an important milestone in CVD risk assessment, as even minimal CAC burden (1-10 Agatston units) is independently associated with a 2-fold higher risk for all-cause mortality compared to no CAC.^7^ Furthermore, there are limited data describing the heterogeneity in vascular aging and incident CAC between older men and women^8^ even though women have a higher prevalence of CAC = 0 at the same age as men, especially among persons above the age of 75 years.

Prior work in the MESA (Multi-Ethnic Study of Atherosclerosis has demonstrated a lack of association between modifiable traditional CVD risk factors and incident CAC in adults ≥65 years of age.^4^ However, older persons have a much shorter time to conversion from CAC = 0 to CAC >0 compared to younger persons and nearly 50% persons in this age group will develop incident CAC within 10 years.^9^ This suggests that the pathophysiology of atherosclerotic plaque calcification may have differing risk factors of highest importance at distinctive stages of the adult life course and/or that the duration of risk factor exposure is poorly estimated by a single measurement of traditional risk factors in older age.

Identifying the risk factors that most strongly associate with the onset of CAC among older men and women may provide insight into: 1) whether certain mechanisms and pathways are more strongly associated with vascular calcification in older age; and 2) the role of nontraditional risk markers in guiding the timing and/or utility of repeat calcium scoring in adults ≥65 years of age. This study aims to prospectively assess the associations of nontraditional CVD risk markers including serum biomarkers of subclinical CVD and extracoronary atherosclerosis with incident CAC among older persons in order to further understand the chronobiology of atherosclerosis and relative contribution of novel risk factors.

## Methods

### Study population

MESA is a community-based prospective cohort study. The specific details on its design and rationale have previously been reported.^10^ Briefly, MESA enrolled 6,814 adults age 45 to 84 years who were free of known clinical CVD, including White, African American, Hispanic, and Chinese participants. All MESA participants had CAC scoring performed at the baseline visit (2000-2002). CAC scoring was performed at subsequent follow-up periods, including MESA Visit 2 (2002-2004), 3 (2004-2005), 4 (2005-2007), and 5 (2010-2011). Due to the MESA study design, not all participants had follow-up CAC scans at each visit. Among persons with CAC = 0 at Visit 1, one-half received a follow-up scan at Visit 2, and the other one-half at Visit 3. Participants who did not have a follow-up CAC scan were prioritized to undergo repeat scanning at Visit 4. Visit 5 included approximately one-half of participants who had CAC = 0 on prior visits, including Visits 3 and 4.^9^ For the current analysis, we included the 815 participants ≥65 years of age who had CAC = 0 at baseline (MESA Visit 1) and a subsequent follow-up CAC scan (MESA Visits 2-5) (Supplemental Figure 1). All variables, including traditional and nontraditional risk factors were measured at study baseline (MESA Visit 1).

All study participants provided written informed consent at each study visit and study protocols were approved at each MESA participating institution and sponsored by the National Heart, Lung, and Blood Institute.

### Measurement of CAC, thoracic aortic calcium, and carotid plaque

Half of the MESA field centers used electron beam computed tomography (MESA: Chicago, Los Angeles, New York), while the other half used multidetector computed tomography (MESA: Baltimore, Forsyth County, St. Paul) to measure CAC.^11^ CAC scores derived from electron beam computed tomography and multidetector computed tomography scanners have excellent agreement (interobserver κ = 0.93 and intraobserver κ = 0.90).^7^ Using the Agatston method, the ascending aorta (aortic annulus to the lower edge of pulmonary artery) and descending aorta (lower edge of pulmonary artery to the cardiac apex) were evaluated for the presence (Agatston score ≥1) or absence (Agatston score = 0) of thoracic aortic calcification (TAC). Of note, TAC was measured at study baseline (Visit 1) for the current investigation. Incident CAC was defined as the presence of CAC >0 at the first follow-up visit (MESA Visit 2 (2002-2004), 3 (2004-2005), 4 (2005-2007), and/or 5 (2010-2011) in which CAC >0 was present.

Baseline (Visit 1) carotid intima-media thickness was evaluated via ultrasound using an M12 L transducer (General Electric Medical Systems; common carotid artery frequency, 13 MHz). The near and far walls of the right and left distal common carotid artery, carotid bulb, and proximal internal carotid artery were examined. The presence of a carotid artery atherosclerotic plaque was defined by a distinct, focal wall thickening ≥1.5 mm or focal thickening ≥50% than the surrounding common carotid intima-media thickness. Carotid plaque measurements were available on a subset (n = 607) of the sample.

### General clinical examination and measurement of ASCVD risk factors

Standardized survey methods were used to collect demographic and clinical information, including sex, race/ethnicity, education status (post high school education vs high school education or less), income (≥$50,000 vs <$50,000 per year), smoking status, and medication use history.^10^ Smoking status was defined as current vs noncurrent smoking.

For blood pressure assessment, blood pressure was measured in triplicate from the brachial artery while participants were in a seated resting position, and the average of the second and third readings. Hypertension was defined as a systolic blood pressure (SBP) >130 mm Hg, diastolic blood pressure >80 mm Hg, or the use of antihypertensive medication. For the calculation of right and left ankle-brachial indices, SBP measurements were obtained from both the upper and lower extremities bilaterally while participants were in a seated resting position using a hand-held Doppler instrument and 5-mHz probe. Specifically, SBP was measured from the brachial, dorsalis pedis, and posterior tibial arteries. The highest of the brachial artery pressures was used as the denominator, whereas the highest pressure from the dorsalis pedis or posterior tibial artery was used for the numerator. Participants were classified as normal (>0.9 and ≤1.3) or abnormal (≤0.9 or >1.3).

Fasting blood glucose was measured using a hexokinase/glucose-6-phosphate dehydrogenase method. Type 2 diabetes was defined as a fasting blood glucose concentration ≥126 mg/dL or the use of glucose-lowering medications. Total cholesterol and high-density lipoprotein cholesterol (HDL-C) were measured enzymatically, and low-density lipoprotein cholesterol values were calculated using the Friedewald equation. An elevated lipoprotein ratio was defined as a total cholesterol/HDL-C ≥3.5 or the use of lipid-lowering medications. Non-HDL-C was calculated as the difference between total cholesterol and HDL-C. Elevated non-HDL-C was defined as a value >130 mg/dL or the use of lipid-lowering medications. Fasting plasma triglycerides were quantified using a glycerol-blanked enzymatic method. Elevated triglycerides were defined as ≥150 mg/dL.

Apolipoprotein-B (Apo-B) was measured using a Tina-quant Apo-B immunoassay on a Roche Modular P analyzer (Roche Diagnostics). Elevated Apo-B was defined as a value >130 mg/dL. Lipoprotein a, Lp(a), concentration was quantified using a latex-enhanced turbidimetric immunoassay (Denka Seiken). Elevated Lp(a) was defined as ≥50 mg/dL for White, Hispanic, and Chinese and ≥30 mg/dL for African American participants. Serum levels of high-sensitivity C-reactive protein (CRP) were measured using the BNIII nephelometer (Dade Behring). Elevated CRP was defined as ≥2 mg/L. Cardiac biomarkers, N-terminal pro-brain natriuretic peptide (NT-proBNP), and high-sensitivity cardiac troponin T (hs-cTnT) were quantified in EDTA plasma and were measured on the Cobas e601(Roche Diagnostics, Indianapolis, IN). Elevated NT-proBNP was defined as ≥125 pg/mL and elevated hs-cTnT was defined ≥3 ng/mL. Measurements of Apo-B (n = 573), Lp(a) (n = 573), and NT-proBNP (n = 662) were available in a subset of the sample.

Serum creatinine was quantified using the Kinetic Jaffe method and was used to calculate estimated glomerular filtration rate (eGFR) via the CKD-EPI equation. An eGFR cutoff of <60 mL/min/1.73 m^2^ was used to define abnormal kidney function (chronic kidney disease stage 2 or higher). A spot urine sample was collected to measure urine albumin using nephelometry and urine creatinine with the Jaffe method. A urine albumin-creatinine ratio of ≥30 mg/g was used to define the presence or absence of albuminuria.

Mediterranean diet was assessed using the 10-point Mediterranean diet score developed by Trichopoulo et al.^12^ Similar to previous studies,^13^ healthy diet was defined by a Mediterranean diet score of >6. Optimal physical activity was defined by >150 minutes of moderate or >75 minutes of vigorous physical activity per week.

### Statistical analyses

Study sample characteristics were presented as mean ± SD for continuous variables, and categorical variables were presented as percentages. Continuous variables that were not normally distributed were presented as median (Q1, Q3). Descriptive statistics were presented for the overall sample and stratified by those with persistent CAC = 0 vs incident CAC. Differences between persistent CAC = 0 and incident CAC groups for normally and nonnormally distributed variables were assessed through the Student’s *t*-test and Wilcoxon signed-rank test, respectively. Differences between categorical variables for those with persistent CAC = 0 vs incident CAC were evaluated through the chi-square test.

Incident CAC was defined as the presence of CAC >0 at the first MESA Visit after Visit 1: MESA Visit 2 (2002-2004), 3 (2004-2005), 4 (2005-2007), and/or 5 (2010-2011). We calculated the proportion with incident CAC in the overall sample and among individuals with elevated nontraditional risk factors. The associations of nontraditional CVD risk factors with incident CAC were assessed through multivariable-adjusted Cox proportional hazards regression. Each nontraditional CVD risk factor was assessed independently in models using categorical variables and continuous (per SD change) variables after adjustment for the following traditional risk factors: age, SBP, diastolic blood pressure, total cholesterol/HDL-C, fasting blood glucose, fasting plasma triglycerides, waist circumference, cigarette smoking, antihypertensive medication, lipid-lowering medication, and glucose-lowering medication (eg, Apo-B per SD change, per 10 mg/dL higher, and at a cutoff value of ≥130 mg/dL). All clinically relevant cutoff values used are included in Supplemental Table 1. We also performed sub-distribution HR modeling to examine for the potential influence of competing risks due to CVD events. Effect modification was examined for the association of nontraditional risk factors with incident CAC according to gender.

To assess the discrimination ability of nontraditional CVD risk factors, we assessed model discrimination through calculated concordance statistics in multivariable Cox proportional hazards regression models. The proportional hazards assumption was satisfied and was tested by assessing the significance of time-dependent independent variables concurrently. Differences in concordance statistics between models were assessed through approaches developed by Uno et al.^14^ The base model for calculating concordance statistics included age, sex, and race. We then evaluated the magnitude of C-statistic improvement when adding the following: 1) all traditional CVD risk factors; 2) individual nontraditional risk factors that were significantly associated with incident CAC; and 3) all nontraditional risk factors that were significantly associated with incident CAC. For comparative purposes, we also calculated the concordance statistic for a bivariate model including the pooled cohort equations (PCE) 10-year ASCVD risk calculator. We conducted a sensitivity analysis excluding individuals on lipid-lowering therapy.

Statistical analyses were performed using SAS 9.3 (SAS Institute). All hypothesis tests were 2-sided. Similar to previous studies that assessed risk factors with CAC outcomes,^15^^,^^16^ we used an alpha threshold <0.05 for detecting differences in descriptive statistics and for detecting significant HRs in Cox proportional hazards regression models.

## Results

**Figure 1:**
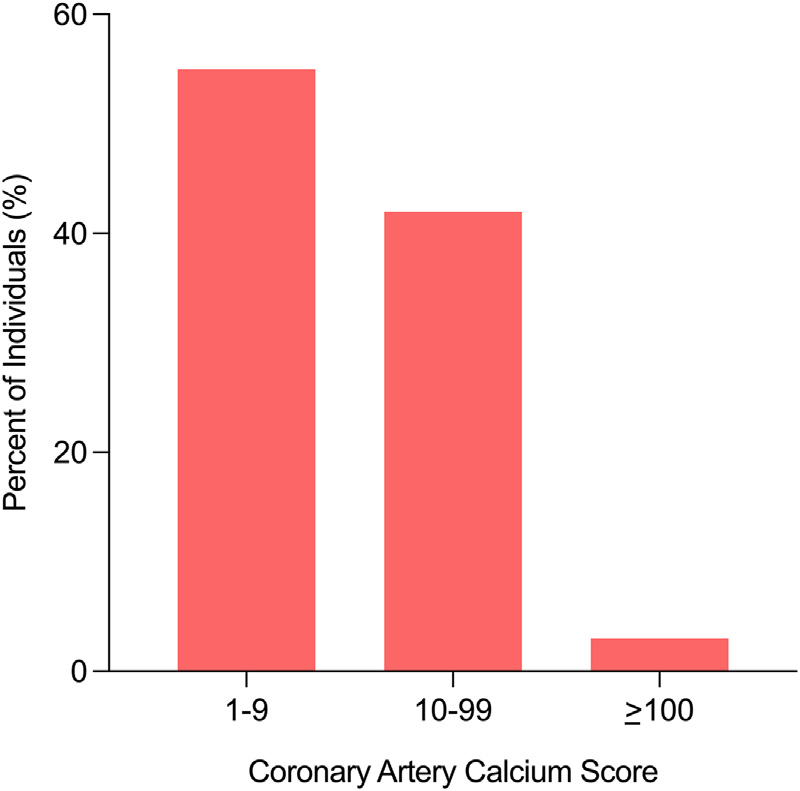
**Distribution of Coronary Artery Calcium Scores Among Persons With Incident CAC** Among older persons that developed incident CAC, the median CAC score was 8 and more than 90% had CAC scores <100. CAC = coronary artery calcium.

The mean age was 70.2 years and 67% were women. A total of 13% of individuals had type 2 diabetes, 18% were prescribed lipid-lowering therapy, and the median 10-year PCE ASCVD risk was 14%. The overall median follow-up time to repeat CAC scan was 3.6 years (IQR: 2.6-9.2 years) and a total of 364 older persons (45%) developed CAC with a median time to incident CAC of 4.3 years. At follow-up, among older persons who developed incident CAC, the median CAC score was 8 and more than 90% had CAC scores <100 (Figure 1). Compared to those with persistent CAC = 0, older persons who developed incident CAC were more likely to be men, have lower HDL-C, higher waist circumference, were more likely to have extracoronary atherosclerosis, and more likely to be on lipid-lowering medication (Table 1). There was no significant difference in age between those who developed incident CAC vs persons who had persistent CAC = 0.

**Table 1:** Characteristics of 815 MESA Primary Prevention Participants >65 Years of Age With Baseline CAC = 0 Who Had a Follow-Up CAC Scan

	All (N = 815)	Persistent CAC = 0 (n = 451)	Incident CAC (n = 364)	*P* Value
Sociodemographic				
Age, y	70.2 ± 4.5	70.4 ± 4.6	70.1 ± 4.3	0.35
Female, %	66.9	70.1	62.9	0.03
Race, %				0.07
White	30.9	27.3	35.4	
Chinese	12.4	13.5	11.0	
African American	35.3	37.7	32.4	
Hispanic	21.4	21.5	21.2	
Post-high school education, %	54.5	53.0	56.3	0.34
Traditional ASCVD risk factors				
Systolic blood pressure, mm Hg	131.1 ± 22.2	130.6 ± 21.8	131.6 ± 22.6	0.52
Diastolic blood pressure, mm Hg	70.6 ± 10.5	70.0 ± 11.0	71.3 ± 10.0	0.09
Antihypertensive medication, %	38.0	36.8	39.6	0.42
Total cholesterol, mg/dL	192.7 ± 33.5	191.9 ± 33.4	193.6 ± 33.6	0.47
LDL-cholesterol, mg/dL	113.9 ± 29.9	112.4 ± 30.4	115.6 ± 29.3	0.14
HDL-cholesterol, mg/dL	55.1 ± 15.3	56.3 ± 15.7	53.6 ± 14.7	0.01
Non-HDL-cholesterol, mg/dL	137.6 ± 32.8	135.7 ± 32.7	140.0 ± 32.7	0.06
Serum triglycerides, mg/dL	104.0 (74.0-147.0)	100.0 (71.0-139.0)	108.0 (77.5-154.0)	0.06
Lipid-lowering medication, %	17.9	14.9	21.7	0.01
Type 2 diabetes, %	12.8	12.0	13.7	0.45
Fasting blood glucose, mg/dL	91.0 (84.0-100.0)	90.0 (83.0-99.0)	92.0 (84.0-102.0)	0.10
Glucose-lowering medication, mg/dL	9.8	8.7	11.0	0.31
Waist circumference, cm	97.2 ± 13.6	95.7 ± 14.0	99.1 ± 13.0	<0.001
Current cigarette smoking, %	5.8	6.0	5.5	0.76
10-y ASCVD risk, %	14.6 (9.3-22.4)	14.1 (8.5-22.5)	15.0 (10.1-22.3)	0.18
Subclinical atherosclerosis				
CAC score at follow-up, AU^a^	0 (0-7)	0 (0-0)	8 (3-21)	<0.001
Presence of thoracic calcification, %	28.6	25.1	33.0	0.01
Presence of carotid plaque, %^b^	33.1	29.0	38.0	0.009
Ankle-brachial index	1.1 ± 0.1	1.1 ± 0.1	1.1 ± 0.1	0.16
Nontraditional ASCVD risk factors				
eGFR, mL/min/1.73 m^2^	72.8 ± 14.1	72.3 ± 14.0	73.4 ± 14.3	0.30
Urine albumin-creatinine ratio, mg/g	6.3 (3.7-12.7)	6.0 (3.6-11.4)	7.0 (3.8-14.4)	0.06
Lipoprotein(a), mg/dL^b^	19.5 (9.4-44.1)	20.4 (9.7-40.0)	19.1 (9.2-47.6)	0.84
Apolipoprotein-B, mg/dL^b^	105.2 ± 26.5	103.4 ± 27.3	107.9 ± 25.1	0.04
hs-cardiac troponin T, ng/mL	4.7 (3.0-7.4)	4.4 (3.0-7.3)	5.2 (3.0-7.5)	<0.001
NT-proBNP, mg/dL^b^	76.0 (40.8-143.0)	74.6 (42.8-136.5)	77.2 (40.0-151.0)	0.64
C-reactive protein, mg/L	2.0 (0.9-3.9)	1.8 (0.9-3.9)	2.1 (0.9-3.9)	0.61
Diet and physical activity				
Healthy diet, %	44.5	40.1	49.7	0.007
Optimal physical activity, %	10.5	11.3	9.6	

Values are mean ± SD, %, or median (IQR).

ASCVD = atherosclerotic cardiovascular disease; CAC = coronary artery calcium; eGFR = estimated glomerular filtration rate; HDL = high-density lipoprotein; LDL = low-density lipoprotein; MESA = Multi-Ethnic Study of Atherosclerosis; NT-proBNP = N-terminal pro-brain natriuretic peptide.

a Reflects CAC score at the initial visit where CAC >1 (Visit 2, 3, 4, or 5).

b Apolipoprotein-B, n = 573; carotid plaque, n = 770; lipoprotein(a), n = 573; NT-proBNP, n = 662.

**Figure 2:**
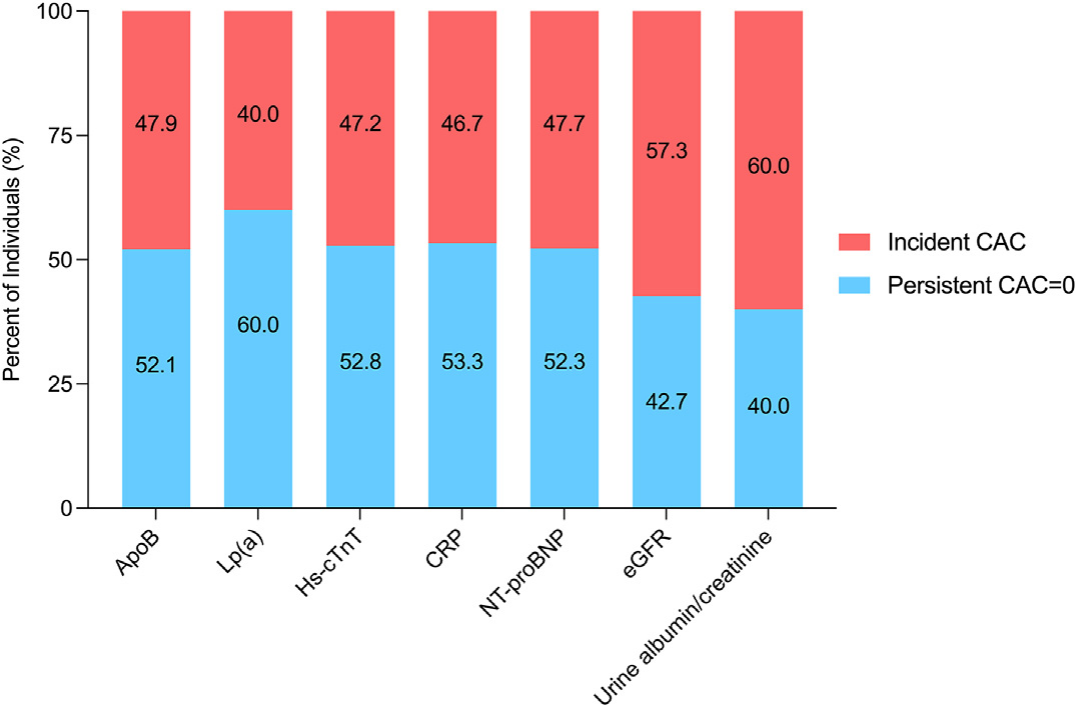
**Proportion of Incident CAC in Persons With Elevated Nontraditional Risk Factors and Serum Biomarkers** Among older individuals with elevated nontraditional risk markers, the highest proportion of individuals with incident CAC was observed for those with albuminuria and an eGFR <60 mL/min/1.73 m^2^, whereas those with elevated Apo-B, hs-cTnT, NT-proBNP, and CRP all had similar but lower proportions of incident CAC. Apo-B = apolipoprotein-B; CAC = coronary artery calcium; CRP = C-reactive protein; eGFR = estimated glomerular filtration rate; hs-cTnT = high-sensitivity cardiac troponin T; Lp(a) = lipoprotein a; NT-proBNP = N-terminal pro-brain natriuretic peptide.

**Figure 3:**
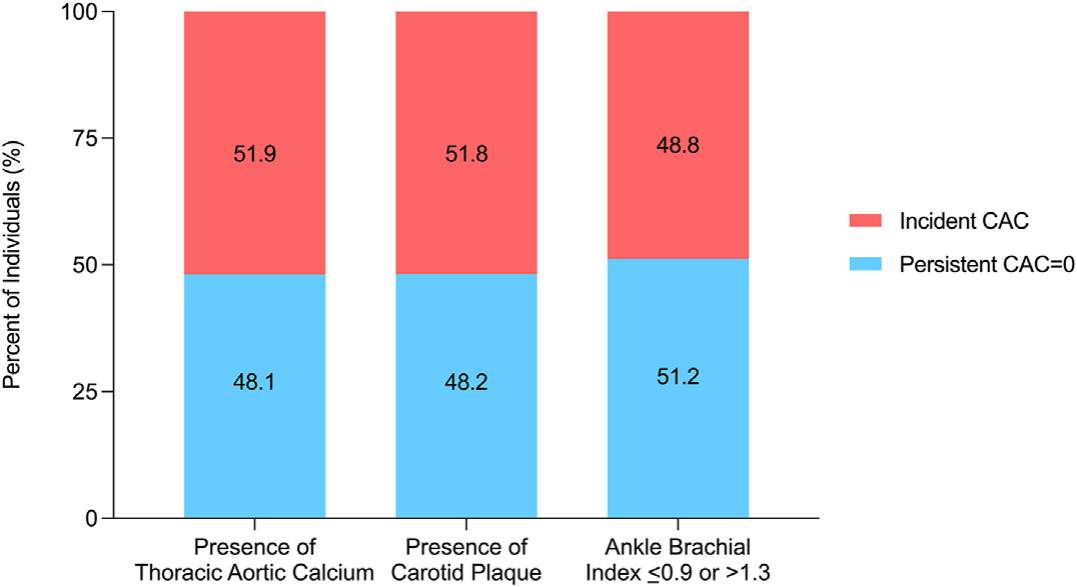
**Proportion of Incident CAC in Persons With Extracoronary Atherosclerosis** There was a higher incidence of CAC for older persons with carotid plaque and TAC (55% and 51%) compared to those with an abnormal ABI (49%). CAC = coronary artery calcium; TAC = thoracic aortic calcification.

Older individuals with incident CAC were more likely to have higher Apo-B and hs-cTnT compared to those with persistent CAC = 0. Mean or median values of other nontraditional risk factors did not significantly differ between CAC groups in descriptive analyses. Among older individuals with elevated nontraditional risk markers, the highest proportion of individuals with incident CAC was observed for those with urine albumin-creatinine ratio ≥30 mg/g (60%) and an eGFR <60 mL/min/1.73 m^2^ (57%), whereas those with elevated Apo-B, hs-cTnT, NT-proBNP, and hsCRP all had similar but lower proportions (47-48%) of incident CAC (Figure 2). The incidence of CAC for older persons with carotid plaque (52%), TAC, and abnormal ABI (49%) was overall similar (Figure 3).

In multivariable modeling, a 1-U SD change in eGFR (HR: 1.19, 95% CI: 1.05-1.34) and the urine albumin-creatinine ratio (HR: 1.17, 95% CI: 1.08-1.26) conferred a 19% and 17% higher risk for incident CAC, respectively (Table 2). SD increment changes in Apo-B, Lp(a), hs-cTnT, and CRP were not significantly associated with incident CAC after the adjustment of traditional ASCVD risk factors in main or sex-stratified analyses. Apo-B and Lp(a) were not significantly associated with incident CAC after removing total cholesterol from multivariable modeling (Supplemental Table 2), and formal collinearity testing among total cholesterol, Apo-B, and Lp(a) was statistically nonsignificant. A significant association between 1-U SD higher NT-proBNP and incident CAC was observed in men but not in women, which was the only risk factor with a consistent difference between men and women for both continuous and categorical calculations.

**Table 2:** Association of Nontraditional ASCVD Risk Factors (per SD Increase) With Incident CAC

Risk Factor^a^	All (N = 815)		Men (n = 270)		Women (n = 545)	
	HR (95% CI)	*P* Value	HR (95% CI)	*P* Value	HR (95% CI)	*P* Value
Lipid						
Apo-B, per 10 mg/dL higher	0.91 (0.65-1.28)	0.60	0.77 (0.36-1.66)	0.51	0.95 (0.64-1.43)	0.82
Lp(a), per 10 mg/dL higher	1.00 (0.88-1.14)	0.99	1.17 (0.89-1.54)	0.26	0.94 (0.80-1.09)	0.41
Myocardial						
Hs-cTnT, per 6 ng/mL higher	1.03 (0.88-1.21)	0.69	1.07 (0.89-1.30)	0.47	1.00 (0.78-1.30)	0.98
NT-proBNP, per 163 mg/dL higher	1.10 (0.99-1.22)	0.08	1.22 (1.08-1.39)	0.002	1.10 (0.87-1.17)	0.91
Renal						
eGFR, per 14 mL/min/1.73 m^2^ lower	1.19 (1.05-1.34)	0.005	0.87 (0.70-1.08)	0.21	1.36 (1.17-1.58)	<0.001
Urinary albumin-creatinine ratio, per 168 mg/g higher	1.17 (1.08-1.26)	<0.001	1.15 (1.05-1.26)	0.003	1.23 (0.99-1.53)	0.06
Inflammatory						
CRP, per 6 mg/L higher	1.03 (0.93-1.15)	0.57	0.88 (0.62-1.24)	0.46	1.10 (0.98-1.23)	0.10

Apo-B = apolipoprotein-B; ASCVD = atherosclerotic cardiovascular disease; CAC = coronary artery calcium; CRP = C-reactive protein; eGFR = estimated glomerular filtration rate; hs-cTnT = high-sensitivity cardiac troponin T; Lp(a) = lipoprotein a; NT-proBNP = N-terminal pro-brain natriuretic peptide.

a Each nontraditional risk factor was evaluated independently after adjustment for age, systolic blood pressure, diastolic blood pressure, total cholesterol, HDL-cholesterol, fasting blood glucose, fasting serum triglycerides, waist circumference, cigarette smoking, antihypertensive medication, lipid-lowering medication, and glucose-lowering medication

In categorical models, a urine albumin-creatinine ratio ≥30 mg/g (HR: 1.50, 95% CI: 1.07-2.09) was consistently associated with incident CAC for men and women, but an eGFR <60 mL/min/1.73 m^2^ was not (Table 3). Similar to models in which the risk factors were examined continuously, nontraditional ASCVD risk markers, when categorized, were not significantly associated with incident CAC in either men or women, except for elevated NT-proBNP at a cutoff of 125 mg/dL in men (HR: 2.07, 95% CI: 1.18-3.65). Among extracoronary atherosclerosis risk factors, presence of carotid plaque (HR: 1.32, 95% CI: 1.04-1.65) and/or TAC (HR: 1.38, 95% CI: 1.10-2.75) significantly associated with incident CAC, whereas an abnormal ankle brachial index did not. When considering competing risks due to CVD events, no significant differences were observed when calculating sub-distribution HRs for incident CAC for urine albumin-creatinine ratio ≥30 mg/g (subdistribution hazard ratio [SHR]: 1.46, 95% CI: 1.04-2.06), carotid plaque (SHR: 1.30, 95% CI: 1.03-1.64), or TAC (SHR: 1.41, 95% CI: 1.11-1.78). Adherence to a Mediterranean diet was associated with higher hazard of incident CAC in women (HR: 1.58, 95% CI: 1.17-2.15) but not men (HR: 0.89, 95% CI: 0.61-1.29). No significant association was observed between optimal physical activity and incident CAC (HR: 0.81, 95% CI: 0.56-1.18).

**Table 3:** Association of Elevated Levels of Novel ASCVD Risk Markers and Presence of Extracoronary Atherosclerosis With Incident CAC

Risk Marker^a^	All (N = 815)		Men (n = 270)		Women (n = 545)	
	HR (95% CI)	*P* Value	HR (95% CI)	*P* Value	HR (95% CI)	*P* Value
Atherogenic						
Apo-B >130 mg/dL	1.00 (0.70-1.44)	0.98	1.11 (0.60-2.03)	0.75	1.17 (0.73-1.87)	0.50
Elevated Lp(a)^b^	0.82 (0.59-1.14)	0.24	0.90 (0.50-1.60)	0.71	0.74 (0.49-1.12)	0.15
Myocardial						
Hs-cTnT >3 ng/mL	1.05 (0.81-1.35)	0.73	1.52 (0.85-2.74)	0.16	0.94 (0.70-1.26)	0.67
NT-proBNP >125 mg/dL	1.19 (0.90-1.60)	0.22	2.07 (1.18-3.65)	0.01	1.01 (0.73-1.41)	0.94
Renal						
eGFR <60 mL/min/1.73 m^2^	0.86 (0.64-1.14)	0.28	1.20 (0.72-2.01)	0.48	0.75 (0.53-1.06)	0.11
Urinary albumin-creatinine ratio >30 mg/g	1.50 (1.07-2.09)	0.01	1.56 (0.94-2.60)	0.09	1.44 (0.92-2.27)	0.11
Inflammatory						
CRP >2 mg/L	1.02 (0.82-1.28)	0.85	0.78 (0.52-1.17)	0.24	1.24 (0.93-1.67)	0.15
Extracoronary atherosclerosis						
Presence of carotid plaque	1.32 (1.04-1.66)	0.02	1.12 (0.75-1.68)	0.57	1.28 (0.95-1.72)	0.10
Presence of thoracic calcification	1.38 (1.10-1.75)	0.006	1.28 (0.80-2.06)	0.31	1.43 (1.08-1.89)	0.01
Ankle-brachial index <0.9 or >1.3	1.02 (0.64-1.62)	0.94	1.20 (0.63-2.29)	0.58	0.82 (0.40-1.68)	0.59
Diet and lifestyle						
Healthy diet (yes vs no)	1.27 (1.01-1.59)	0.04	0.89 (0.61-1.29)	0.53	1.58 (1.17-2.15)	0.003
Optimal physical activity (yes vs no)	0.81 (0.56-1.18)	0.28	0.94 (0.55-1.59)	0.81	0.70 (0.40-1.20)	0.19

Apo-B = apolipoprotein-B; ASCVD = atherosclerotic cardiovascular disease; CAC = coronary artery calcium; CRP = C-reactive protein; eGFR = estimated glomerular filtration rate; hs-cTnT = high-sensitivity cardiac troponin T; Lp(a) = lipoprotein a; NT-proBNP = N-terminal pro-brain natriuretic peptide.

a Each nontraditional risk factor was evaluated independently after adjustment for age, hypertension, total cholesterol/HDL-cholesterol, type 2 diabetes, hypertriglyceridemia, abdominal obesity, cigarette smoking, antihypertensive medication, lipid-lowering medication, and glucose-lowering medication

b Lp(a) >50 mg/dL for non-Hispanic White, Hispanic, and Chinese, Lp(a) >30 mg/dL for non-Hispanic Black.

When added to demographic information, urine albumin-creatinine ratio, carotid plaque, and TAC were significantly associated with incident CAC and provided a larger magnitude improvement in the C-statistic for incident CAC discrimination compared all traditional ASCVD risk factors combined (Table 4, Central Illustration). The addition of the urine albumin-creatinine ratio (C-statistic = 0.604, Δ+0.037, *P* = 0.02), carotid plaque (C-statistic = 0.605, Δ+0.038, *P* = 0.02), TAC (C-statistic = 0.609, Δ+0.042, *P* = 0.01), and all combinations of the urine albumin-creatinine ratio, carotid plaque, and TAC provided significant improvements in discrimination for incident CAC discrimination, whereas traditional risk factors and the PCE risk score did not. When considering a model with all 3 significant risk factors, including urine albumin-creatinine ratio, carotid plaque, and TAC, there was a +0.047 magnitude higher C-statistic (C-statistic = 0.614, *P* = 0.004) for the discrimination of incident CAC compared to demographic information alone. There was no significant difference in the discrimination of incident CAC when urine albumin-creatinine ratio, carotid plaque, and TAC were added to traditional risk factors (Δ+0.014, *P* = 0.09).

**Table 4:** AUC Analysis for Conversion to CAC >0 Among Older Persons, Stratified by Sex

	All (N = 815)			Men (n = 270)			Women (n = 545)		
	C-Statistic	ΔC-Statistic	ΔC-Statistic *P* Value	C-Statistic	ΔC-Statistic	ΔC-Statistic *P* Value	C-Statistic	ΔC-Statistic	ΔC-Statistic *P* Value
Demographics									
Age, sex, race	0.567	-	-	0.563	-	-	0.557	-	-
Traditional risk factors									
Traditional risk factors^a^	0.600	+0.033	0.05	0.609	+0.042	0.09	0.601	+0.044	0.08
Pooled cohort equations ASCVD risk score	0.567	0.000	0.94	0.524	−0.039	0.49	0.562	+0.005	0.80
Novel risk factors and imaging									
Individual risk factors + urine albumin-creatinine ratio	0.604	+0.037	0.02	0.615	+0.052	0.04	0.601	+0.044	0.07
Individual risk factors + carotid plaque	0.605	+0.038	0.02	0.614	+0.051	0.05	0.605	+0.048	0.04
Individual risk factors + TAC	0.609	+0.042	0.01	0.611	+0.048	0.08	0.611	+0.054	0.04
Individual risk factors + urine albumin-creatinine ratio + carotid plaque	0.609	+0.042	0.01	0.617	+0.054	0.03	0.609	+0.052	0.03
Individual risk factors + urine albumin-creatinine ratio + TAC	0.613	+0.046	0.006	0.617	+0.054	0.05	0.611	+0.054	0.04
Individual risk factors + carotid plaque + TAC	0.614	+0.047	0.007	0.618	+0.055	0.03	0.614	+0.057	0.08
Individual risk factors + urine albumin-creatinine ratio + carotid plaque + TAC	0.614	+0.047	0.004	0.618	+0.055	0.04	0.614	+0.057	0.03

All models included age, sex, and race.

ASCVD = atherosclerotic cardiovascular disease; AUC = area under the curve; CAC = coronary artery calcium; TAC = thoracic aortic calcification.

a Cigarette smoking, systolic blood pressure, diastolic blood pressure, total cholesterol, high-density lipoprotein cholesterol, blood glucose, triglycerides, waist circumference, antihypertensive medication, lipid-lowering medication, glucose-lowering medication.

**Central Illustration:**
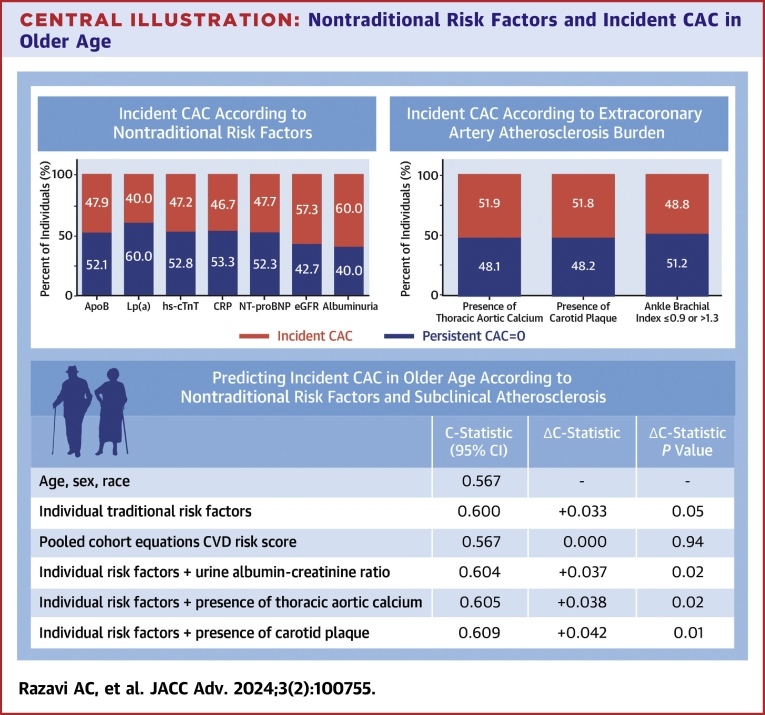
**Nontraditional Risk Factors and Incident CAC in Older Age** Nearly one-half of persons ≥65 years of age developed incident CAC over a median follow-up time of <4 years. Among nontraditional RF and measures of subclinical CVD or atherosclerosis, only albuminuria and TAC were significantly associated with incident CAC in persons ≥65 years of age. Apo-B = apolipoprotein-B; CAC = coronary artery calcium; CRP = C-reactive protein; CVD = cardiovascular disease; eGFR = estimated glomerular filtration rate; hs-cTnT = high-sensitivity cardiac troponin T; Lp(a) = lipoprotein a; NT-proBNP = N-terminal pro-brain natriuretic peptide; TAC = thoracic aortic calcification.

Multivariable regression and discrimination statistics were consistent after excluding individuals on lipid-lowering when compared to the main study sample results (Supplemental Tables 3 to 5).

## Discussion

Beyond traditional risk factors, we found that the urine albumin-creatinine ratio, carotid plaque, and TAC were significantly associated with incident CAC and also improved the discrimination of incident CAC, whereas nontraditional lipid, inflammatory, and myocardial risk markers did not. Furthermore, the addition of the urine albumin-creatinine ratio, carotid plaque, and/or TAC to demographic information provided larger magnitude improvements for the detection of incident CAC when compared to all traditional CVD risk factors combined, the latter of which did not significantly improve incident CAC discrimination when added to information on age, sex, and ethnicity. These results demonstrate that early detection of nephropathy and extracoronary atherosclerosis are important risk factors among older persons, which may help guide the utility and timing of repeat CAC scoring in older persons with baseline CAC = 0.

The main clinical implication of our findings relates to approaches for CVD risk stratification in older adults who have some form of atherosclerotic resilience. While individuals who have persistent CAC = 0 through older age represent a unique and low-risk group,^17^ we found that nearly one-half developed incident CAC with a median time to conversion of 3 to 4 years, which is associated with a significant increase in the risk for ASCVD.^18^ Given the relative suboptimal performance of traditional risk factors and the PCE risk score for CVD risk stratification in older compared to younger persons,^19^ our results provide timely information with respect to the utility of nontraditional risk factors in guiding repeat CAC scoring. Older persons with albuminuria and/or TAC may represent a higher risk subgroup among those with CAC = 0 who would benefit from more aggressive lifestyle interventions, closer monitoring of risk factors, and shorter duration between repeat CAC scoring.

Older individuals who maintain CAC = 0 even in the presence of risk factors appear to represent a unique, healthy subgroup that should be undergo different CVD risk stratification protocols vs those with prevalent CAC. Nevertheless, the presence of CAC = 0 should not encourage refraining from global risk factor control because control and/or elimination of risk factors that only modestly improve model performance can still yield considerable reductions in population-level CVD events.^20^ While the duration of risk factor exposure is important to consider when identifying CAC = 0 clinically, genetics and lifestyle habits are also likely contributors to the manifestation and maintenance of this resilient phenotype.

The urine albumin-creatinine ratio was consistently associated with incident CAC on the continuous scale and at the most commonly used albuminuria cutoff of 30 mg/g. Specifically, we found that older individuals with albuminuria had a 54% higher risk of incident CAC compared to those without albuminuria, independent of traditional CVD risk factors. These results extend on previous literature that shows a stepwise increase in CAC across increasing urinary albumin excretion thresholds among individuals with prevalent subclinical atherosclerosis, which in part appears to be mediated by hypertension and type 2 diabetes.^21^ Given our findings and the low laboratory costs of urine albumin testing, our results suggest that routine measurement and incorporation of albuminuria within CVD risk assessment among adults ≥65 years of age who have CAC = 0 may be a useful strategy to personalize timing for repeat CAC scoring.

We also found that the presence of carotid plaque and/or TAC conferred between a 35% to 38% higher risk for incident CAC among older individuals. Contrastingly, abnormal ABI did not significantly associate with incident CAC among older individuals after adjustment for traditional risk factors. The lack of association between abnormal ABI and incident CAC may be because ABI is a surrogate measure of subclinical atherosclerosis, whereas quantification of carotid plaque on ultrasound and TAC on cardiac CT are direct measures of subclinical atherosclerosis burden. The associations of carotid plaque and TAC with incident CAC were approximately 1.5-fold stronger in women compared to men, and this finding extends upon current data showing that a majority of women are more likely to develop extracoronary atherosclerosis prior to CAC.^22^ Our findings suggest that identification of prevalent carotid plaque and/or TAC in older persons with CAC = 0 may prompt consideration of repeat calcium scoring on cardiac computed tomography, even among older persons.

We identified significant associations between NT-proBNP and incident CAC in men but not women without heart failure at baseline. Subclinical elevations in NT-proBNP among individuals without clinical CVD are a known risk factor for developing myocardial dysfunction,^23^ but few studies have identified an association with CAC specifically. Previous studies^24^ have been inconsistent with respect to identifying sex differences in the prognostic utility of NT-proBNP in ASCVD risk stratification and whether it can improve risk beyond traditional risk factors. Further research is needed to determine if there truly is a difference for NT-proBNP and the initiation of CAC among men vs women.

Our results did not show a significant association between atherogenic lipids and incident CAC, and nonsignificant associations of Apo-B and Lp(a) with incident CAC in persons ≥65 years of age persisted even after removing total cholesterol from multivariable models. Though our findings showed that Lp(a) and Apo-B did not independently associate with incident CAC, lipids are still an important part of the atherosclerotic process that should be monitored and treated in appropriately selected patients, including older individuals who maintain CAC = 0. Our findings may also support recent research which shows that low-density lipoprotein cholesterol is predominantly associated with CVD risk among those with CAC >0 but not CAC = 0.^25^ Overall, our negative results underline the challenges in identifying factors associated with subclinical atherosclerosis in older persons and suggest potential different mechanisms of vascular calcification across the life course.

The main strength of the current research includes a comprehensive assessment of nontraditional risk markers for incident CAC in older persons across atherogenic, inflammatory, myocardial, and subclinical atherosclerosis mechanisms. While there currently remain limited data regarding the utility of repeat CAC scoring in older persons, our study provides timely information that may aid in the consideration of repeat CAC scoring among older individuals according to nontraditional risk factor burden.

Our study should be interpreted in the setting of certain limitations, most importantly survival bias. We included individuals aged ≥65 years who had baseline CAC = 0, who are a population much healthier than persons of similar age in the United States. Related to survival bias is the limitation in characterizing risk in older persons, such that the lowering of CVD risk through treatment of risk factors (eg, Apo-B) does not necessarily correspond to the risk factors themselves strongly associating with subclinical atherosclerosis and clinical outcomes. Moreover, the median follow-up time was 3.6 years, and our sample size was relatively small (n = 815), though a longer follow-up period with more participants would have been beneficial to detect a potential higher incidence of CAC. Therefore, we may have had a lower power to detect significant associations between nontraditional risk factors and incident CAC. However, a large proportion of participants had been preselected to obtain follow-up CAC scans at Visit 5 according to the MESA design. While an additional limitation is that we do not know the exact time of conversion to CAC >0, the very low scores observed among those that did develop incident CAC and the multiple follow-up visits suggests that our follow-up time estimates are not dramatically different from the exact time of CAC onset. Our statistical approaches were also inferential and not predictive, which may limit our conclusions regarding the utility of nontraditional risk factors for incident CAC prediction in older adults. We were unable to control for duration of exposure to risk factors prior to the start of the MESA study or after the baseline visit, which may have further improved precision and reduced residual confounding when identifying significant nontraditional risk markers associated with incident CAC. Lastly, while we used a *P* value of <0.05 to test for statistical significance, we did not perform testing for multiple comparisons and therefore the results should be interpreted in this context.

In conclusion, albuminuria, carotid plaque, and TAC were significantly associated with incident CAC in persons ≥65 years of age beyond nontraditional risk markers, but other nontraditional risk factors were not. Early detection of the urine albumin-creatinine ratio and extracoronary atherosclerosis may help guide the utility and timing of repeat CAC scoring in older persons with baseline CAC = 0.

PERSPECTIVES **COMPETENCY IN MEDICAL KNOWLEDGE:** Nearly one-half of persons ≥65 years of age developed incident CAC over a median-follow up time of <4 years. Among nontraditional RF and measures of subclinical CVD or atherosclerosis, only albuminuria and TAC were significantly associated with incident CAC in persons ≥65 years of age. Identification of older persons with albuminuria or TAC may help guide the utility and timing of repeat CAC scoring in older persons with baseline CAC = 0.

**TRANSLATIONAL OUTLOOK:** Future and large prospective studies with longer follow-up period between CAC scans are required to comprehensively identify the nontraditional risk factors that can help guide the optimal timing of repeat CAC scoring and treatment allocation of preventive therapies in primary prevention patients ≥65 years of age.

## Funding support and author disclosures

This research was supported by R01 HL071739 and contracts 75N92020D00001, HHSN268201500003I. MESA was supported by contracts N01-HC-95159, 75N92020D00005, N01-HC-95160, 75N92020D00002, N01-HC-95161, 75N92020D00003, N01-HC-95162, 75N92020D00006, N01-HC-95163, 75N92020D00004, N01-HC-95164, 75N92020D00007, N01-HC-95165, N01- HC-95166, N01-HC-95167, N01-HC-95168, and N01-HC- 95169 from the National Heart, Lung, and Blood Institute, and by grants UL1-TR-000040, UL1-TR-001079, and UL1-TR-001420 from the National Center for Advancing Translational Sciences (NCATS). Dr Blaha has received grants from the National Institutes of Health, U.S. Food and Drug Administration, American Heart Association, and Aetna Foundation; grants and personal fees from Amgen; and personal fees from Sanofi, Regeneron, Novartis, Bayer, and Novo Nordisk outside the submitted work. Dr Michos is on advisory boards of AstraZeneca, Bayer, Boehringer Ingelheim, Esperion, Novo Nordisk, Novartis, and Pfizer. All other authors have reported that they have no relationships relevant to the contents of this paper to disclose.
