# Supplementary material for: Nontraditional Risk Markers for Incident Coronary Artery Calcium Among Persons ≥65 Years of Age
Source: JACC Adv. 2023 Dec 19;3(2):100755. doi: 10.1016/j.jacadv.2023.100755 (PMC11198298; doi:10.1016/j.jacadv.2023.100755)

| **Supplemental Table 1.** | |
| --- | --- |
| **Variable** | **Cutoff Value** |
| **Traditional Risk Factors** |  |
| Type 2 Diabetes | Fasting blood glucose >126 mg/dL or the use of glucose-lowering medications |
| Elevated lipoprotein ratio | Total cholesterol/HDL-C >3.5 or the use of lipid-lowering medication |
| Elevated non-HDL-C | Non-HDL-C >130 mg/dL or the use of lipid-lowering medication |
| Elevated triglycerides | Triglycerides >150 mg/dL |
| Carotid Plaque | >1.5 mm or a focal thickening >50% than the surrounding common carotid intima-media thickness |
| Abnormal ABI | <0.9 or 1.3 |
| **Non-Traditional Risk Factors** |  |
| Elevated ApoB | ApoB >130 mg/dL |
| Elevated Lp(a) | >50 mg/dL for White, Hispanic, and Chinese and >30 mg/dL for African American participants |
| Elevated CRP | >2 mg/L |
| Elevated NT-proBNP | >125 mg/dL |
| Elevated hs-cTnT | >3 ng/mL |
| Abnormal renal function | <60 mL/min/1.73 m^2^ |
| Albuminuria | >30 mg/g |

| **Supplemental Table 2.** Association of ApoB and Lp(a) with Incident CAC without Adjusting for Total Cholesterol | | |
| --- | --- | --- |
| **Risk Factor** | **HR (95% CI)** | **P-Value** |
| **ApoB*** |  |  |
| Elevated ApoB | 1.08 (0.76, 1.53) | 0.68 |
| ApoB (per SD higher) | 1.11 (0.97, 1.28) | 0.13 |
|  |  |  |
| **Lp(a)*** |  |  |
| Elevated Lp(a) | 0.83 (0.60, 1.15) | 0.26 |
| Lp(a) (per SD higher) | 1.02 (0.90, 1.16) | 0.80 |
| *adjusted for age, systolic blood pressure, diastolic blood pressure, HDL-cholesterol fasting blood glucose, fasting serum triglycerides, waist circumference, cigarette smoking, antihypertensive medication, lipid-lowering medication, and glucose-lowering medication | | |

| **Supplemental Table 3**. Association Non-Traditional ASCVD Risk Factors (per SD increase) with Incident CAC, Excluding Individuals on Lipid-Lowering Medication | | | | | | |
| --- | --- | --- | --- | --- | --- | --- |
|  | **All (n=658)** | | **Men (n=223)** | | **Women (n=445)** | |
| **Risk Factor*** | **Hazard Ratio (95% CI)** | **P-Value** | **Hazard Ratio (95% CI)** | **P-Value** | **Hazard Ratio (95% CI)** | **P-Value** |
| Lipid |  |  |  |  |  |  |
| ApoB, per 10 mg/dL higher | 0.91 (0.65, 1.28) | 0.60 | 0.77 (0.36, 1.66) | 0.51 | 0.95 (0.64, 1.43) | 0.82 |
| Lp(a), per 10 mg/dL higher | 1.00 (0.88, 1.14) | 0.99 | 1.17 (0.89, 1.54) | 0.26 | 0.94 (0.80, 1.09) | 0.41 |
| Myocardial |  |  |  |  |  |  |
| Hs-cTnT, per 6 ng/mL higher | 1.03 (0.87, 1.22) | 0.72 | 1.09 (0.91, 1.31) | 0.36 | 0.99 (0.74, 1.31) | 0.93 |
| NT-proBNP, per 163 mg/dL higher | 1.11 (0.99, 1.24) | 0.07 | 1.17 (1.101, 1.36) | 0.03 | 1.01 (0.85, 1.20) | 0.94 |
| Renal |  |  |  |  |  |  |
| eGFR, per 14 mL/min/1.73 m^2^ lower | 1.21 (1.06, 1.39) | 0.006 | 0.88 (0.69, 1.11) | 0.27 | 1.42 (1.19, 1.70) | <0.001 |
| Urinary Albumin-Creatinine Ratio, per 168 mg/g higher | 1.15 (1.06, 1.25) | <0.001 | 1.15 (1.04, 1.27) | 0.007 | 1.18 (0.93, 1.48) | 0.17 |
| Inflammatory |  |  |  |  |  |  |
| CRP, per 6 mg/L higher | 1.04 (0.93, 1.15) | 0.52 | 0.93 (0.67, 1.28) | 0.64 | 1.11 (0.99, 1.25) | 0.09 |
| *each non-traditional risk factor was evaluated independently after adjustment for age, systolic blood pressure, diastolic blood pressure, fasting blood glucose, fasting serum triglycerides, waist circumference, cigarette smoking, antihypertensive medication, and glucose-lowering medication | | | | | | |

| **Supplemental Table 4**. Association of Elevated levels of Novel ASCVD Risk Markers and Presence of Extra-Coronary Atherosclerosis with Incident CAC | | | | | | |
| --- | --- | --- | --- | --- | --- | --- |
|  | **All (n=658)** | | **Men (n=223)** | | **Women (n=445)** | |
| **Risk Marker*** | **Hazard Ratio (95% CI)** | **P-Value** | **Hazard Ratio (95% CI)** | **P-Value** | **Hazard Ratio (95% CI)** | **P-Value** |
| Atherogenic |  |  |  |  |  |  |
| ApoB >130 mg/dL | 1.00 (0.70, 1.44) | 0.98 | 1.11 (0.60, 2.03) | 0.75 | 1.17 (0.73, 1.87) | 0.50 |
| Elevated Lp(a) † | 0.82 (0.59, 1.14) | 0.24 | 0.90 (0.50, 1.60) | 0.71 | 0.74 (0.49, 1.12) | 0.15 |
| Myocardial |  |  |  |  |  |  |
| Hs-cTnT >3 ng/mL | 1.14 (0.85, 1.54) | 0.37 | 2.19 (1.10, 4.35) | 0.03 | 0.97 (0.69, 1.36) | 0.86 |
| NT-proBNP >125 mg/dL | 1.22 (0.88, 1.69) | 0.23 | 1.75 (0.94, 3.26) | 0.08 | 1.03 (0.70, 1.52) | 0.87 |
| Renal |  |  |  |  |  |  |
| eGFR <60 mL/min/1.73 m^2^ | 0.76 (0.54, 1.06) | 0.11 | 0.94 (0.52, 1.71) | 0.84 | 0.67 (0.45, 1.02) | 0.06 |
| Urinary Albumin-Creatinine Ratio >30 mg/g | 1.68 (1.15, 2.47) | 0.008 | 1.81 (1.02, 3.20) | 0.04 | 1.65 (0.96, 2.83) | 0.07 |
| Inflammatory |  |  |  |  |  |  |
| CRP >2 mg/L | 1.01 (0.79, 1.31) | 0.92 | 0.81 (0.53, 1.26) | 0.35 | 1.27 (0.90, 1.79) | 0.18 |
| Extra-Coronary Atherosclerosis |  |  |  |  |  |  |
| Presence of Carotid Plaque | 1.34 (1.04, 1.74) | 0.03 | 1.03 (0.65, 1.63) | 0.90 | 1.42 (1.02, 1.97) | 0.03 |
| Presence of Thoracic Calcification | 1.60 (1.22, 2.10) | <0.001 | 1.30 (0.76, 2.24) | 0.34 | 1.78 (1.28, 2.49) | <0.001 |
| Ankle-Brachial Index <0.9 or >1.3 | 1.13 (0.68, 1.90) | 0.64 | 1.00 (0.49, 2.03) | 0.99 | 1.22 (0.55, 2.69) | 0.62 |
| *each non-traditional risk factor was evaluated independently after adjustment for age, hypertension, total cholesterol/HDL-cholesterol, type 2 diabetes, hypertriglyceridemia, abdominal obesity, cigarette smoking, antihypertensive medication, lipid-lowering medication, and glucose-lowering medication  †Lp(a) >50 mg/dL for non-Hispanic white, Hispanic, and Chinese, Lp(a) >30 mg/dL for non-Hispanic black. | | | | | | |

| **Supplemental Table 5**. AUC Analysis for Conversion to CAC >0 Among Older Persons After Excluding Individuals on Lipid-Lowering Medication, Stratified by Sex | | | | | | | | | |
| --- | --- | --- | --- | --- | --- | --- | --- | --- | --- |
|  | **All (n=658)** | | | **Men (n=223)** | | | **Women (n=445)** | | |
|  | **C-Statistic** | **ΔC-Statistic** | **ΔC-Statistic P-Value** | **C-Statistic** | **ΔC-Statistic** | **ΔC-Statistic P-Value** | **C-Statistic** | **ΔC-Statistic** | **ΔC-Statistic P-Value** |
| Demographics |  |  |  |  |  |  |  |  |  |
| Age, sex, race | 0.579 | - | - | 0.574 | - | - | 0.574 | - | - |
| Traditional Risk Factors |  |  |  |  |  |  |  |  |  |
| Individual risk factors* | 0.599 | +0.200 | 0.34 | 0.614 | +0.040 | 0.07 | 0.608 | +0.034 | 0.10 |
| Pooled cohort equations ASCVD risk score | 0.566 | -0.013 | 0.61 | 0.525 | -0.049 | 0.47 | 0.566 | -0.008 | 0.79 |
| Novel Risk Factors and Imaging |  |  |  |  |  |  |  |  |  |
| Individual risk factors + urine albumin-creatinine ratio | 0.604 | +0.025 | 0.23 | 0.638 | +0.064 | 0.06 | 0.608 | +0.034 | 0.10 |
| Individual risk factors + carotid plaque | 0.605 | +0.024 | 0.21 | 0.640 | +0.066 | 0.06 | 0.619 | +0.045 | 0.04 |
| Individual risk factors + TAC | 0.613 | +0.034 | 0.10 | 0.637 | +0.063 | 0.07 | 0.624 | +0.050 | 0.09 |
| Individual risk factors + urine albumin-creatinine ratio +  carotid plaque | 0.610 | +0.031 | 0.14 | 0.638 | +0.064 | 0.06 | 0.622 | +0.048 | 0.04 |
| Individual risk factors + urine albumin-creatinine ratio +  TAC | 0.617 | +0.038 | 0.06 | 0.638 | +0.064 | 0.06 | 0.627 | +0.053 | 0.08 |
| Individuals risk factors + carotid plaque + TAC | 0.621 | +0.042 | 0.07 | 0.638 | +0.064 | 0.07 | 0.635 | +0.061 | 0.04 |
| Individual risk factors + urine albumin-creatinine ratio + carotid plaque + TAC | 0.621 | +0.042 | 0.04 | 0.636 | +0.062 | 0.05 | 0.638 | +0.064 | 0.04 |
| *cigarette smoking, systolic blood pressure, diastolic blood pressure, total cholesterol, HDL-C, blood glucose, triglycerides, waist circumference, antihypertensive medication, glucose-lowering medication | | | | | | | | | |

**Supplemental Figure 1**. Percentage of participants with follow-up CAC scans according to MESA study Visit.


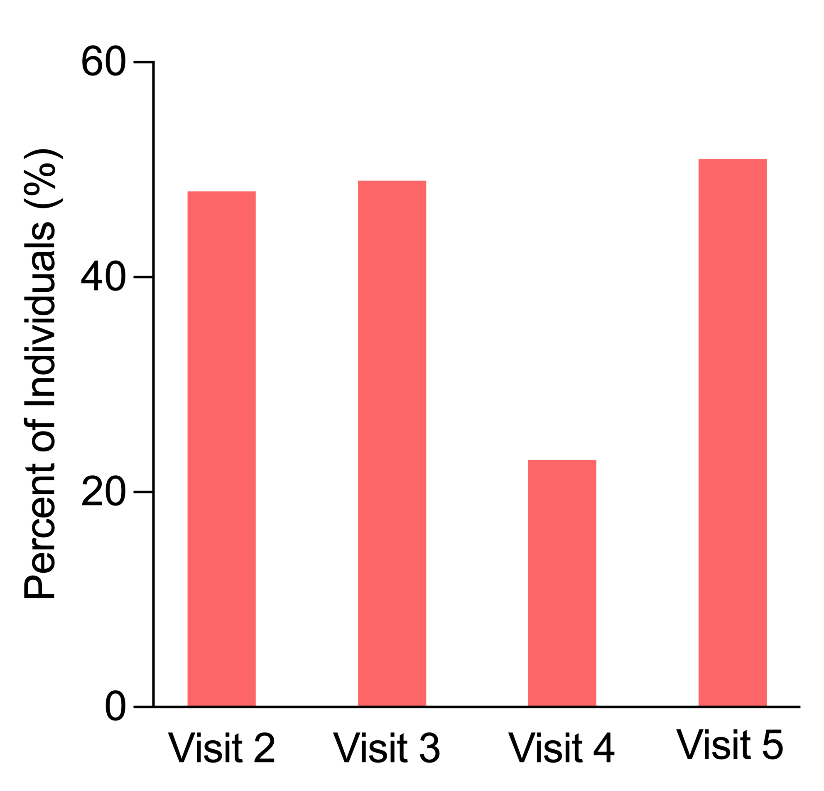

Supplement: Supplemental Tables 1-5 and Figure 1 [file mmc1.docx]
